# Surface EMG-based quantification of inspiratory effort: a quantitative comparison with *P*_es_

**DOI:** 10.1186/s13054-021-03833-w

**Published:** 2021-12-20

**Authors:** Jan Graßhoff, Eike Petersen, Franziska Farquharson, Max Kustermann, Hans-Joachim Kabitz, Philipp Rostalski, Stephan Walterspacher

**Affiliations:** 1 Institute for Electrical Engineering in Medicine, Universität zu Lübeck, Moislinger Allee 53-55, 23558 Lübeck, Germany; 2 Fraunhofer Research Institution for Individualized and Cell-Based Medical Engineering, Mönkhofer Weg 239 a, 23562 Lübeck, Germany; 3 Medical Clinic II, Klinikum Konstanz, Mainaustraße 35, 78464 Konstanz, Germany; 4 Faculty of Health/School of Medicine, Witten/Herdecke University, Alfred-Herrhausen-Straße 50, 58455 Witten, Germany

**Keywords:** Assisted mechanical ventilation, Inspiratory effort, Monitoring, Esophageal pressure, Surface electromyography

## Abstract

**Background:**

Inspiratory patient effort under assisted mechanical ventilation is an important quantity for assessing patient–ventilator interaction and recognizing over and under assistance. An established clinical standard is respiratory muscle pressure $\textit{P}_{\mathrm{mus}}$, derived from esophageal pressure ($\textit{P}_{\mathrm{es}}$), which requires the correct placement and calibration of an esophageal balloon catheter. Surface electromyography (sEMG) of the respiratory muscles represents a promising and straightforward alternative technique, enabling non-invasive monitoring of patient activity.

**Methods:**

A prospective observational study was conducted with patients under assisted mechanical ventilation, who were scheduled for elective bronchoscopy. Airway flow and pressure, esophageal/gastric pressures and sEMG of the diaphragm and intercostal muscles were recorded at four levels of pressure support ventilation. Patient efforts were quantified via the $\textit{P}_{\mathrm{mus}}$-time product (${\mathrm{PTP}}_{\mathrm{mus}}$), the transdiaphragmatic pressure-time product (${\mathrm{PTP}}_{\mathrm{di}}$) and the EMG-time products (ETP) of the two sEMG channels. To improve the signal-to-noise ratio, a method for automatically selecting the more informative of the sEMG channels was investigated. Correlation between ETP and ${\mathrm{PTP}}_{\mathrm{mus}}$ was assessed by determining a neuromechanical conversion factor $\textit{K}_{\mathrm{EMG}}$ between the two quantities. Moreover, it was investigated whether this scalar can be reliably determined from airway pressure during occlusion maneuvers, thus allowing to quantify inspiratory effort based solely on sEMG measurements.

**Results:**

In total, 62 patients with heterogeneous pulmonary diseases were enrolled in the study, 43 of which were included in the data analysis. The ETP of the two sEMG channels was well correlated with ${\mathrm{PTP}}_{\mathrm{mus}}$ ($\textit{r}={0.79\pm 0.25}$ and $\textit{r}={0.84\pm 0.16}$ for diaphragm and intercostal recordings, respectively). The proposed automatic channel selection method improved correlation with ${\mathrm{PTP}}_{\mathrm{mus}}$ ($\textit{r}={0.87\pm 0.09}$). The neuromechanical conversion factor obtained by fitting ETP to ${\mathrm{PTP}}_{\mathrm{mus}}$ varied widely between patients ($\textit{K}_{\mathrm{EMG}}= {4.32\pm 3.73}\,{\hbox {cm}\hbox {H}_{2}\hbox {O}/\upmu \hbox {V}}$) and was highly correlated with the scalar determined during occlusions ($\textit{r}={0.95}$, $\textit{p}<{.001}$). The occlusion-based method for deriving ${\mathrm{PTP}}_{\mathrm{mus}}$ from ETP showed a breath-wise deviation to ${\mathrm{PTP}}_{\mathrm{mus}}$ of ${0.43\pm 1.73}\,{\hbox {cm}\hbox {H}_{2}\hbox {O}\,\hbox {s}}$ across all datasets.

**Conclusion:**

These results support the use of surface electromyography as a non-invasive alternative for monitoring breath-by-breath inspiratory effort of patients under assisted mechanical ventilation.

**Supplementary Information:**

The online version contains supplementary material available at 10.1186/s13054-021-03833-w.

## Introduction

In assisted mechanical ventilation, the work of breathing is shared between patient and ventilator. Excessive assistance, resulting in diaphragmatic dysfunction and patient–ventilator asynchrony, as well as insufficient assistance, leading to diaphragmatic fatigue, should be avoided [1]. In view of this, a new paradigm has been introduced, termed *diaphragm-protective ventilation*, advocating to closely monitor spontaneous breathing effort and to adjust ventilator settings such that an adequate division of the respiratory workload is reached [2–4]. A current clinical standard for quantifying inspiratory effort is to measure esophageal pressure $P_{\mathrm {es}}$ and then derive an estimate of respiratory muscle pressure $P_{\mathrm {mus}}$ from this measurement, mainly by correcting for the influence of the chest wall elastance [3, 5]. Measuring $P_{\mathrm {es}}$ requires the positioning of an esophageal balloon catheter with adequate filling volumes [6]. Despite its usefulness, $P_{\mathrm {es}}$ is still not frequently used in many clinics due to a number of practical drawbacks [7].

In recent years, the invasive measurement of the electrical activity of the diaphragm ($\mathrm {EAdi}$) has been increasingly embraced as a potential alternative to $P_{\mathrm {es}}$ for monitoring respiratory effort [1–4, 8]. This signal is also obtained using an esophageal catheter, which, instead of a balloon, is equipped with concentric ring electrodes to measure the electrical fields generated by the diaphragm during contraction [9, 10]. Contrary to the $P_{\mathrm {es}}$ signal, which measures the indirect results of force generation performed by the respiratory muscles, $\mathrm {EAdi}$ directly reflects the neural drive to the diaphragm muscle [11]. To derive an estimate of $P_{\mathrm {mus}}$ from $\mathrm {EAdi}$, Bellani et al. [12] calculated a $P_{\mathrm {mus}}/\mathrm {EAdi}$ index during occlusion maneuvers. They found $\mathrm {EAdi}$ and $P_{\mathrm {mus}}$ to be closely correlated within patients and the ratio of the two measures to be stable across different ventilation modes and assistance levels. This enables pneumatic estimates of a patient’s inspiratory effort to be obtained from $\mathrm {EAdi}$, requiring occlusions as a calibration maneuver.

In a number of publications, *surface electromyography (sEMG)*—sometimes also called *transcutaneous* EMG—has been proposed as a completely non-invasive alternative for monitoring the efforts of some or all inspiratory and expiratory muscles by means of electrodes placed on the skin surface [13–19]. Besides the utility of sEMG measurements for monitoring patient–ventilator asynchrony [20, 21], first attempts have been made for estimating $P_{\mathrm {mus}}$ based on sEMG measurements. As with $\mathrm {EAdi}$, there is a patient- and muscle-specific conversion factor that relates the level of sEMG measured above a muscle to the force or pressure generated by that muscle. Similar to their earlier study on $\mathrm {EAdi}$, Bellani et al. [22] identified a $P_{\mathrm {mus}}/\mathrm {sEMG}$ conversion factor during occlusion maneuvers and found the resulting $\mathrm {sEMG}$-based estimate of $P_{\mathrm {mus}}$ to be closely correlated with $P_{\mathrm {mus}}$ derived from $P_{\mathrm {es}}$ when aggregating multiple similar breaths. After aggregation of breaths, they also found a high degree of correlation between $\mathrm {sEMG}$ and $\mathrm {EAdi}$.

In this article, building upon previous sEMG-related studies [22], we investigate the relationship between the respiratory sEMG signals and $P_{\mathrm {mus}}$ (as well as transdiaphragmatic pressure $P_{\mathrm {di}}$) derived from esophageal/gastric pressure measurements. To this end, we analyze study data of patients under assisted mechanical ventilation with endotracheal intubation, who were scheduled for elective bronchoscopy. Our main objective is to investigate the estimation of the $P_{\mathrm {mus}}$ pressure-time product (PTP) via sEMG by identifying a patient-specific conversion factor during end-expiratory occlusions. As a measure for the sEMG-derived inspiratory effort, we use the EMG-time product (ETP) which is calculated as the integral of the EMG curve against an adaptive baseline. Moreover, we propose and test a novel channel selection method to leverage the benefit of multiple sEMG measurement channels being available. As opposed to previous studies on respiratory sEMG, we also investigate the linearity of the sEMG-$P_{\mathrm {mus}}$ relation and the quantification of breath-wise efforts without relying on aggregation.

## Methods

### Patients

The study was conducted at the department of pneumology, cardiology and intensive care of the Klinikum Konstanz (Konstanz, Germany) and registered in the German Clinical Trials Register (DRKS00021524). The protocol was approved by the ethics committee of Witten/Herdecke University (Witten, Germany) and conducted in adherence to the ethical standards laid down in the Declaration of Helsinki in its most current form. Patients older than 18 years scheduled for elective bronchoscopy under mechanical ventilation using flexible endotracheal tubes were enrolled for the study; exclusion criteria were pregnancy, severe obesity, neuromuscular disorders, drug abuse, bleeding diathesis and contraindication for placement of a nasogastric catheter (esophageal stenosis and esophageal varices). Signed informed consent was obtained from patients prior inclusion to the study.

### Measurements

A nasogastric double-balloon catheter (Bösch, Gottenheim, Germany) was filled according to the recommendations in [6], and esophageal/gastric pressures ($P_{\mathrm {es}}$, $P_{\mathrm {ga}}$) were measured with pressure transducers connected to the proximal end of the catheter. The correct positioning of the esophageal balloon was confirmed via the airway occlusion technique [23]. The surface EMG was measured using two pairs of pre-gelled Ag/AgCl electrodes at the following positions: bilaterally at the lower costal margin on the midclavicular line and bilaterally in the second intercostal space on the parasternal line [14, 24]. A common electrode was placed above the sternum. The sEMG signals were amplified and recorded at a sampling rate of ${1000} \, \hbox {Hz}$ using a dedicated amplifier and acquisition software provided by Dräger (Drägerwerk AG & Co. KGaA, Lübeck, Germany). The device was also used to digitize and record the analog signals from the pressure transducers ($P_{\mathrm {es}}$ and $P_{\mathrm {ga}}$) at a sampling rate of ${200}\, \hbox {Hz}$ (sEMG Base, Drägerwerk AG & Co. KGaA, Lübeck, Germany). The airway flow ($\dot{V}$) and pressure ($P_{\mathrm {aw}}$) tracings from the Dräger V500 ventilator (Drägerwerk AG & Co. KGaA, Lübeck, Germany) were acquired through the ventilator’s RS232 interface at ${100}\, \hbox {Hz}$ and then synchronized with the remaining signals.

### Study protocol

After patients were enrolled in the study, they were intubated and put on assisted spontaneous ventilation using a sedation protocol with propofol. All patients were sedated to a level of moderate/deep sedation corresponding to level −3 to −4 of the Richmond agitation sedation scale for the study measurement period. Oxygen supplementation was titrated as low as possible to maintain SpO2 of at least 90%. Following the initial positioning of the esophageal balloon, a series of spontaneous inspiratory efforts against occluded airways was recorded. Initially, patients were ventilated with continuous positive airway pressure (CPAP) on a Dräger V500 ventilator (Drägerwerk AG & Co. KGaA, Lübeck, Germany). Patients were then switched to pressure support ventilation (PSV), and three levels of assistance (5, 10 and ${15}\,{\hbox {cm}\hbox {H}_{2}\hbox {O}}$) were applied in random order. Throughout the protocol, a positive end-expiratory pressure (PEEP) of ${5}\,{\hbox {cm}\hbox {H}_{2}\hbox {O}}$ was used.

### Data preprocessing

Segments strongly affected by artifacts (e.g., due to ventilator fighting and coughing) were manually marked as invalid and excluded from the analysis. Similarly, measurement errors and artifacts in $P_{\mathrm {es}}$—e.g., due to peristalsis—were marked and the corresponding signal segments excluded from any analysis involving $P_{\mathrm {es}}$.

#### Processing of $P_{\mathrm {es}}$ and $P_{\mathrm {ga}}$

**Fig. 1:**
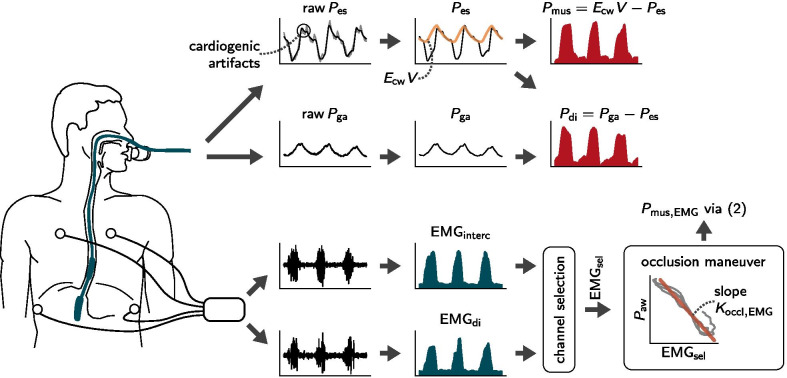
An overview of the processing pipelines for surface EMG and esophageal/gastric pressure signals. Esophageal pressure ($\textit{P}_{\mathrm{es}}$) and gastric pressure ($\textit{P}_{\mathrm{ga}}$) are measured simultaneously with the double balloon technique. Cardiogenic artifacts are removed from the raw pressure signals via template subtraction. The muscular pressure ($\textit{P}_{\mathrm{mus}}$) is then calculated as the difference between $\textit{P}_{\mathrm{es}}$ and the chest wall recoil pressure $\textit{P}_{\mathrm{cw}}$ (orange curve, given by the product of the chest wall elastance $\textit{E}_{\mathrm{cw}}$ and the volume signal $\textit{V}$). Transdiaphragmatic pressure ($\textit{P}_{\mathrm{di}}$) is calculated as the difference between $\textit{P}_{\mathrm{es}}$ and $\textit{P}_{\mathrm{ga}}$ curves. The respiratory surface EMG is measured via two pairs of electrodes positioned bilaterally at the second intercostal space and the costal margin. The envelopes ${\mathrm{EMG}}_{\mathrm{di}}$ and ${\mathrm{EMG}}_{\mathrm{interc}}$ are calculated on the raw ECG-gated signals using a moving RMS filter. Then, the more informative of the two channels, denoted as ${\mathrm{EMG}}_{\mathrm{sel}}$, is automatically selected and fitted to the airway pressure $\textit{P}_{\mathrm{aw}}$ over the course of multiple subsequent occlusions, providing a scalar $\textit{K}_{\mathrm{occl,EMG}}$. The estimate $\textit{P}_{\mathrm{mus,EMG}}$ is calculated via the factor $\textit{K}_{\mathrm{occl,EMG}}$ and a baseline-corrected ${\mathrm{EMG}}_{\mathrm{sel}}$ signal as in Eq. (2)

As the first step towards identifying the pressure $P_{\mathrm {mus}}$ from $P_{\mathrm {es}}$, cardiogenic pressure artifacts were removed from both the $P_{\mathrm {ga}}$ and $P_{\mathrm {es}}$ signals. For this step, a template subtraction method was employed, cf. [25] for details. The time course of the transdiaphragmatic pressure $P_{\mathrm {di}}$ was then calculated as the difference between $P_{\mathrm {ga}}$ and $P_{\mathrm {es}}$. The pressure $P_{\mathrm {mus}}$ generated by the respiratory muscles at each instant was calculated as the difference between esophageal pressure $P_{\mathrm {es}}$ and the elastic recoil of the chest wall $P_\mathrm {cw}=E_{\mathrm {cw}}\cdot V\!.$ To this end, the chest wall elastance $E_{\mathrm {cw}}$ was determined under the highest pressure support level as described in Additional file 1. Figure 1 provides a graphical overview of the processing steps undertaken to estimate $P_{\mathrm {mus}}$ and $P_{\mathrm {di}}$ based on $P_{\mathrm {es}}$.

Next, we used the available end-expiratory occlusions to check and correct the scaling of both $P_{\mathrm {es}}$ and $P_{\mathrm {mus}}$. During occlusions, as flow and volume are zero, the pressure drop in $P_{\mathrm {aw}}$ can be assumed to be equal to $P_{\mathrm {mus}}$ (and the relative drop in $P_{\mathrm {es}}$), which allows assessing possible scaling errors in $P_{\mathrm {es}}$, e.g., due to catheter positioning errors [23]. Thus, following the balloon positioning procedure, we determined a correction factor $K_\mathrm {occl,es}$ by fitting the esophageal pressure waveform to the airway pressure waveform over the course of multiple subsequent occlusions by means of linear regression. In practice, small scaling errors remain even after proper positioning, i.e., the factor $K_\mathrm {occl,es}$ is often slightly larger than one. In the following, we correct for these remaining errors by scaling the $P_{\mathrm {mus}}$ waveform with the factor $K_\mathrm {occl,es}$ we determined during occlusions. More details on the signal preprocessing are provided in Additional file 1.

#### Preprocessing of the sEMG signals

The ECG artifact in the two sEMG channels was removed using a gating technique. The envelopes of the two sEMG channels were then calculated using a moving ${250}\,\hbox {ms}$ RMS filter; the diaphragmatic and the intercostal EMG channels are denoted as $\mathrm {EMG}_\mathrm {di}$ and $\mathrm {EMG}_\mathrm {interc}$, respectively. The envelopes of sEMG measurements often have an offset in the order of several $\upmu \hbox {V}$ due to measurement noise. The level of this offset can be assessed during phases in which the patient is passive, e.g., during expirations. We corrected for these offsets by calculating an adaptive, time-varying baseline value and subtracting it from the envelopes, details are given in Additional file 1. After baseline subtraction, both envelopes were indeed roughly zero when the patient was almost passive.

### Data analysis

#### Effort-time products

We employed the pressure-time product (PTP) as a measure of inspiratory effort because it has been shown to capture patient efforts better than work of breathing (WOB) when little or no volume is generated [5], e.g., due to missed efforts. To calculate PTP, recordings were first segmented into inspirations and expirations using a simple, threshold-based detector that was directly applied to the $P_{\mathrm {mus}}$ signal. The detector was based on the trigger algorithm proposed by Sinderby et al. [26], details are provided in Additional file 1. The breath-wise PTP expressed in $\hbox {cm}\hbox {H}_{2}\hbox {O}\,\hbox {s}$ was then calculated as the area under the $P_{\mathrm {mus}}$ and $P_{\mathrm {di}}$ waveforms over the course of an inspiration. We denote the two resulting quantities by $\mathrm {PTP}_\mathrm {mus}$ and $\mathrm {PTP}_\mathrm {di}$, respectively. Finally, any efforts exceeding $\mathrm {PTP}_\mathrm {mus}={20}\,{\hbox {cm}\hbox {H}_{2}\hbox {O}\,\hbox {s}}$ were excluded from further analyses, because such unusually forceful breaths do not fall into the range of normal tidal breathing targeted in this study and therefore could distort the results. Analogously to PTP, the two EMG-time products $\mathrm {ETP}_\mathrm {di}$ and $\mathrm {ETP}_\mathrm {interc}$, expressed in ${\upmu \hbox {V} \hbox {s}}$, were calculated by breath-wise integration of the inspiratory segment of the two (baseline-adjusted, cf. above) sEMG channels.

#### Channel selection

In many patients, a difference in sEMG amplitudes measured at the intercostal muscles and at the diaphragm can be observed. This difference may be attributed to different breathing patterns, e.g., abdominal or thoracic breathing, but also to differences in skin-electrode impedance or subcutaneous tissue thickness. Often the level of baseline noise differs between the two channels as well. To exploit the availability of multiple measurement channels we investigated a simple, automatic method for selecting the more informative of the two channels based on the signal-to-noise ratio (SNR). The SNR of the two sEMG channels was approximated by forming the ratio between the maximum amplitudes reached during tidal breathing and the amplitude of the measurement noise; details are given in Additional file 1. For each patient, the channel with higher SNR was selected for quantifying inspiratory effort. We denote the selected channel by $\mathrm {EMG}_\mathrm {sel}$, and the corresponding EMG-time product by $\mathrm {ETP}_\mathrm {sel}$. The herein proposed selection method is in contrast to the approach by Bellani et al. [22], who investigated a different channel combination strategy (the addition of available envelopes) which however did not improve results in their study.

#### Neuromechanical conversion factor

In many muscles, an approximately linear relationship has been observed between an appropriately processed surface EMG envelope signal and the force generated by the muscle under observation [27]. Concerning the respiratory muscles, [28] showed that for very high activation levels, the $P_{\mathrm {di}}$-EAdi relation can become nonlinear. In contrast, in ventilated patients no change in neuromechanical coupling was found across a large range of pressure support levels [29]. Similarly, Bellani et al. [12] reported that the $P_{\mathrm {mus}}$-EAdi relation could be well approximated via a linear model within the studied range of respiratory activities and proceeded to use a linear conversion parameter, calling it the “$P_{\mathrm {mus}}$/EAdi index”. Petersen et al. [30] used a linear combination of multiple respiratory sEMG channels to estimate $P_{\mathrm {mus}}$, reporting no improvement when employing nonlinear regression within a physiological range of respiratory loads. Thus, we calculated a linear neuromechanical conversion factor, denoted as $K_\mathrm {EMG}$, between the different ETP metrics and $\mathrm {PTP}_\mathrm {mus}$ by means of regression. This was done by directly fitting the efforts via the linear regression model

1 $$\begin{aligned} \mathrm {PTP}_\mathrm {mus}=K_\mathrm {EMG}\cdot \mathrm {ETP} + P_{\mathrm {bias}}\cdot T_\mathrm {i} \end{aligned}$$

and solving for the unknown parameters $K_\mathrm {EMG}$ and $P_{\mathrm {bias}}$. Here, $P_{\mathrm {bias}}$ is a constant bias term and $T_\mathrm {i}$ is the length of the detected effort, which accounts for the integration of the bias over the duration of each effort. In that sense, the parameter $P_{\mathrm {bias}}$ represents systemic offsets that the EMG envelope might have against the muscular pressure curve $P_{\mathrm {mus}}$.

#### Occlusions

As proposed by Bellani et al. [12, 22], we determined a neuromechanical conversion factor $K_\mathrm {occl,EMG}$ as a surrogate for $K_\mathrm {EMG}$: we fitted the selected sEMG envelope $\mathrm {EMG}_\mathrm {sel}$ to the airway pressure waveform $P_{\mathrm {aw}}$ during multiple subsequent occlusion maneuvers, cf. Fig. 1. The parameter $K_\mathrm {occl,EMG}$ is therefore an approximation to the ‘true’ neuromechanical factor $K_\mathrm {EMG}$, cf. Eq. (1), and is determined completely non-invasively without relying on $P_{\mathrm {es}}$ as a reference. Using $K_\mathrm {occl,EMG}$, expressed as $\hbox {cm}\hbox {H}_{2}\hbox {O}/\upmu \hbox {V}$, a continuous $P_{\mathrm {mus}}$ estimate can be obtained as

2 $$\begin{aligned} P_{\mathrm {mus,EMG}}=\alpha \cdot K_\mathrm {occl,EMG}(\mathrm {EMG}_\mathrm {sel}-\mathrm {EMG}_\mathrm {sel,0}), \end{aligned}$$

where $\mathrm {EMG}_\mathrm {sel,0}$ denotes the EMG baseline and $\alpha$ is a constant correction factor accounting for known systematic overestimation when determining the neuromechanical scalar during occlusions. This overestimation can be attributed to the isometric configuration of the diaphragm muscle in the absence of flow, leading to a higher neuromechanical conversion factor than during normal breathing. The parameter $\alpha$ is intended to correct for this systematic deviation. Numerical values for $\alpha$ were determined on our patient cohort and compared to the proposed values from earlier studies [12, 22]. Using $K_\mathrm {occl,EMG}$, the inspiratory effort was estimated via

3 $$\begin{aligned} \mathrm {PTP}_\mathrm {mus,EMG}= \int P_{\mathrm {mus,EMG}}\,\mathrm {d} t = \alpha \cdot K_\mathrm {occl,EMG}\cdot \mathrm {ETP}_\mathrm {sel}. \end{aligned}$$

#### Statistics

Results are expressed as ${\mathrm{mean} \pm \mathrm{standard~deviation}}$. Correlation between variables was quantified by means of Pearson’s correlation coefficient *r*. Deviations between $\mathrm {PTP}_\mathrm {mus,EMG}$ and $\mathrm {PTP}_\mathrm {mus}$ were analyzed using the Bland-Altman limit of agreements with repeated measures [31], and additionally, the mean absolute deviation (MAD) is reported as an error metric. A two-tailed Wilcoxon signed-rank test was used to identify differences between the channel with the higher SNR, i.e.,  the selected channel and the respective other channel with lower SNR.

## Results

**Fig. 2:**
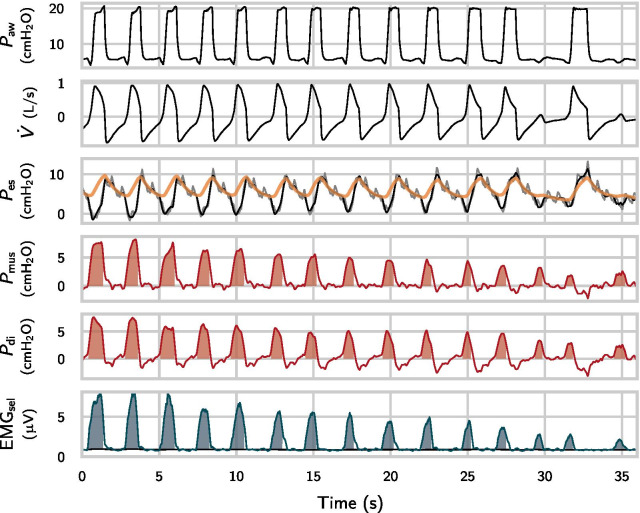
Exemplary excerpt of relevant signals during assisted ventilation. The orange line is the estimated curve for the chest-wall recoil $\textit{E}_{\mathrm{cw}} \textit{V}$ and the grey line is the raw $\textit{P}_{\mathrm{es}}$ signal before removal of artifacts. The envelope of the automatically selected EMG channel is denoted by ${\mathrm{EMG}}_{\mathrm{sel}}$ (green line). The shaded areas correspond to PTP and ETP measures. ETP is calculated against an adaptive baseline (black line in the bottom graph)

A total of 62 patients were enrolled in the study, 43 of which were included in the data analysis. The first nine patients were excluded due to technical issues. Additionally, patients were excluded from the analysis if they met one of the following criteria (respective number of patients given in brackets): failure to employ the esophageal/gastric catheter (2), technical recording issues (1), corrupted $P_{\mathrm {es}}$ signal, e.g., due to balloon positioning issues or $K_\mathrm {occl,es}>2$ (5), and failure of the sEMG cardiac artifact gating algorithm (2). In one patient, $P_{\mathrm {ga}}$ was not available. This patient was therefore not included in the $P_{\mathrm {di}}$-based results. In two patients, no end-expiratory occlusions longer than ${0.35}\,{\hbox {s}}$ were available. These two patients were therefore only included in the correlation analysis but not in the calculation of neuromechanical conversion factors or in the comparison of absolute efforts. Table 1 summarizes the characteristics of the included patients; Fig. 2 shows an exemplary excerpt of a recording.

**Table 1:** Clinical characteristics of patients included in the analysis $(n=43)$

Characteristic	Result
Age, $\hbox {mean} \pm \hbox {SD}$ year	${64\pm 11}$
Men, $\textit{n}$ (%)	34 (79)
Weight, $\hbox {mean} \pm \hbox {SD}$ $\hbox {kg}$	${79.0\pm 8.9}$
BMI, $\hbox {mean} \pm \hbox {SD}$ $\hbox {kg}\,\hbox {m}^{-2}$	${26\pm 6}$
TLC, $\hbox {mean} \pm \hbox {SD}$ l	${6.7\pm 2.4}$
VC, $\hbox {mean} \pm \hbox {SD}$ l	${3.6\pm 1.4}$
FEV1 % predicted, $\hbox {mean} \pm \hbox {SD}$ %	${82\pm 29}$
Tiffeneau index, $\hbox {mean} \pm \hbox {SD}$ %	${69\pm 19}$
RV, $\hbox {mean} \pm \hbox {SD}$ l	${3.4\pm 2.4}$
RV/TLC, $\hbox {mean} \pm \hbox {SD}$ %	${45\pm 19}$
iPEEP, $\hbox {mean} \pm \hbox {SD}$ ${\hbox {cm}\hbox {H}_{2}\hbox {O}}$	${2.1\pm 1.4}$
Diagnosis, $\textit{n}$ (%)	
OSAS	4 (9)
COPD	16 (37)
GOLD I	2 (5)
GOLD II–III	14 (33)
ACOS	3 (7)
Bronchial asthma	5 (19)
ILD	7 (17)
Lung cancer	20 (47)
Infectious or rheumatic diseases	11 (26)

Body-mass index (BMI), total lung capacity (TLC), vital capacity (VC), forced expiratory volume in ${1}\,\hbox {s}$ (FEV1), residual volume (RV), intrinsic PEEP (iPEEP), obstructive sleep apnea syndrome (OSAS), chronic obstructive pulmonary disease (COPD), asthma-COPD overlap syndrome (ACOS), interstitial lung disease (ILD). In several patients, multiple pulmonary/systemic diseases were diagnosed

**Fig. 3:**
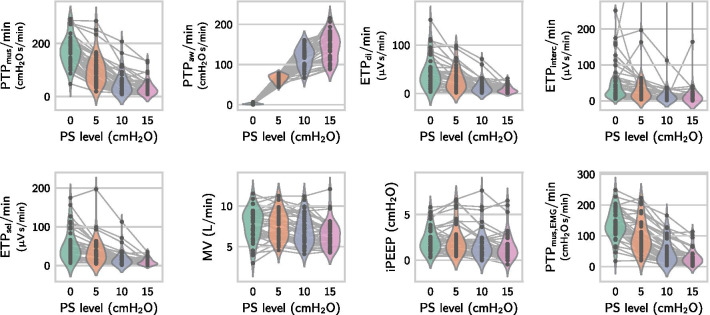
Effect of different pressure support levels on muscular and airway pressure-time products (${\mathrm{PTP}}_{\mathrm{mus}}$ and ${\mathrm{PTP}}_{\mathrm{aw}}$), EMG-time products of the diaphragm, intercostal and selected channel (${\mathrm{ETP}}_{\mathrm{di}}$, ${\mathrm{ETP}}_{\mathrm{interc}}$, ${\mathrm{ETP}}_{\mathrm{sel}}$), minute ventilation (MV), dynamic intrinsic PEEP (iPEEP) and the sEMG-derived estimate (${\mathrm{PTP}}_{\mathrm{mus,EMG}}$). The PTP$/\hbox {min}$ and ETP$/\hbox {min}$ values were calculated by aggregating all efforts in each support level and then dividing by the length of the segment. Each point corresponds to one patient and one pressure support level. In ${\mathrm{ETP}}_{\mathrm{interc}}$, three outliers $>{400}\,{\upmu \hbox {V}\hbox {s}/\hbox {min}}$ from a single patient are not shown within the plotting range. Numerical values (mean ± SD) of the data are reported in Additional file 2

In the 43 patients selected for further analysis, the esophageal scaling factor was found to be close to one ($K_\mathrm {occl,es}={1.18\pm 0.18}$), indicating the validity of the balloon position and filling. The analyzed recording length per patient was ${23.3\pm 4.0}\,{\hbox {min}}$, and the number of detected efforts in each recording was ${454\pm 137}$. Across all 43 patients, a total of 19 540 inspiratory efforts with a length of ${0.89\pm 0.31}\,{\hbox {s}}$ were included for analysis. The average number of analyzed occlusion maneuvers per patient was ${4.8\pm 1.5}$. As expected, the inspiratory effort measured by $\mathrm {PTP}_\mathrm {mus}/\hbox {min}$ and $\mathrm {ETP}_\mathrm {sel}/\hbox {min}$ decreased with higher support levels, while the total ventilator ‘effort’ $\mathrm {PTP}_\mathrm {aw}/\hbox {min}$ increased (Fig. 3). In most patients, intrinsic PEEP was low, except for 11 patients in which we observed a dynamic intrinsic PEEP higher than ${3}\,{\hbox {cm}\hbox {H}_{2}\hbox {O}}$ (measured during CPAP ventilation from the $P_{\mathrm {mus}}$ value required to initiate lung inflation), cf. Table 1 and Fig. 3.

Table 2 provides breath-wise correlations between the different measures of inspiratory efforts in individual patients: we found a good correlation between ETP and $\mathrm {PTP}_\mathrm {mus}$ (and, thus, between $\mathrm {PTP}_\mathrm {mus,EMG}$ and $\mathrm {PTP}_\mathrm {mus}$) as well as $\mathrm {PTP}_\mathrm {di}$. To assess the influence of the SNR-based selection strategy on ETP-$\mathrm {PTP}_\mathrm {mus}$ correlation, we tested for differences between the selected channels and the respective other channels with lower SNR and found a higher correlation with $\mathrm {PTP}_\mathrm {mus}$ in the selected channel ($p=0.029$). In comparison to always using either one of the two EMG channels, the automatic selection method increased the correlation with $\mathrm {PTP}_\mathrm {mus}$ to ${0.87\pm 0.09}$. The estimated $\mathrm {SNR}$ value was ${1.73\pm 0.56}$ for the diaphragm channel and ${1.87\pm 0.94}$ for the intercostal channel. In 25 out of 41 patients, the intercostal channel was selected as the more informative of the two channels.

**Table 2:** Pearson correlation coefficient $\textit{r}$ between different metrics of inspiratory effort, considering all observed breaths in individual patients ($\hbox {mean} \pm \hbox {SD}$), $n= 43$ (and $n=42$ where $\textit{P}_{\mathrm{di}}$ is involved)

	${\mathrm{ETP}}_{\mathrm{interc}}$	${\mathrm{ETP}}_{\mathrm{sel}}$	${\mathrm{PTP}}_{\mathrm{mus,EMG}}$	${\mathrm{PTP}}_{\mathrm{mus}}$	${\mathrm{PTP}}_{\mathrm{di}}$
${\mathrm{ETP}}_{\mathrm{di}}$	${0.74\pm 0.27}$	${0.89\pm 0.16}$	${0.89\pm 0.16}$	${0.84\pm 0.16}$	0.84 ± 0.16
${\mathrm{ETP}}_{\mathrm{interc}}$	–	${0.86\pm 0.28}$	${0.86\pm 0.28}$	${0.79\pm 0.25}$	0.77 ± 0.26
${\mathrm{ETP}}_{\mathrm{sel}}$	$\star$	–	${1.0\pm 0.0}$	${0.87\pm 0.09}$	0.86 ± 0.10
${\mathrm{PTP}}_{\mathrm{mus,EMG}}$	$\star$	$\star$	–	${0.87\pm 0.09}$	0.86 ± 0.10
${\mathrm{PTP}}_{\mathrm{mus}}$	$\star$	$\star$	$\star$	–	0.97 ± 0.05

The included effort metrics are: muscular and transdiaphragmatic pressure-time products (${\mathrm{PTP}}_{\mathrm{mus}}$ and ${\mathrm{PTP}}_{\mathrm{di}}$), EMG-time products of diaphragm, intercostal and selected channel (${\mathrm{ETP}}_{\mathrm{di}}$, ${\mathrm{ETP}}_{\mathrm{interc}}$, ${\mathrm{ETP}}_{\mathrm{sel}}$) and sEMG-derived muscular pressure-time product (${\mathrm{PTP}}_{\mathrm{mus,EMG}}$). Entries marked with $\star$ are given by symmetry.

### Neuromechanical conversion factor $K_\mathrm {EMG}$ and bias

**Fig. 4:**
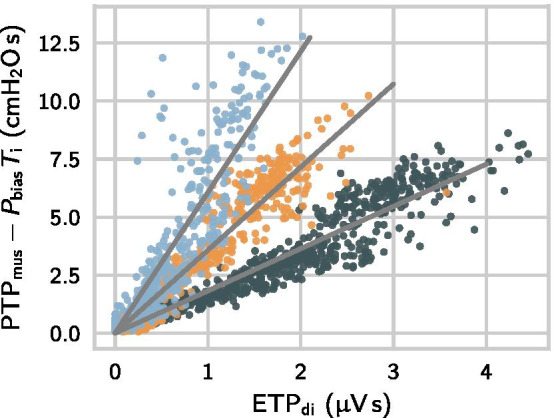
The ${\mathrm{ETP}}_{\mathrm{di}}$–${\mathrm{PTP}}_{\mathrm{mus}}$ relation in three selected patients with $\textit{K}_{\mathrm{EMG}}$ ranging from ${1.8}\,{\hbox {cm}\hbox {H}_{2}\hbox {O}/\upmu \hbox {V}}$ to ${6.1}\,{\hbox {cm}\hbox {H}_{2}\hbox {O}/\upmu \hbox {V}}$. In all three patients, biases were small (the absolute value of $\textit{P}_{\mathrm{bias}}$ was smaller than ${0.6}\,{\hbox {cm}\hbox {H}_{2}\hbox {O}}$) and biases were removed in this plot via the term $\textit{P}_{\mathrm{bias}}\cdot \textit{T}_{\mathrm{i}}$. The correlations between ${\mathrm{ETP}}_{\mathrm{di}}$ and ${\mathrm{PTP}}_{\mathrm{mus}}$ were $\textit{r}= 0.94$ (dark green), $\textit{r}= {0.95}$ (orange) and $\textit{r}= {0.86}$ (blue)

Table 3 provides numerical results on the neuromechanical factor $K_\mathrm {EMG}$ and bias $P_{\mathrm {bias}}$ between different ETP values and $\mathrm {PTP}_\mathrm {mus}$. The neuromechanical factor $K_\mathrm {EMG}$ varied widely between patients, whereas the bias term was small. Figure 4 shows the $\mathrm {ETP}_\mathrm {di}$–$\mathrm {PTP}_\mathrm {mus}$ scatter diagram for three selected patients with strongly varying neuromechanical conversion factors $K_\mathrm {EMG}$. For the automatically selected EMG channel, $K_\mathrm {EMG}$ ranged from ${0.7}\,\hbox {cm}\hbox {H}_{2}\hbox {O}/\upmu \hbox {V}$ to ${16.8}\,\hbox {cm}\hbox {H}_{2}\hbox {O}/\upmu \hbox {V}$. We found a weak positive correlation between the neuromechanical factors calculated for the intercostal and the diaphragm channels ($r=0.38$, $p=.014$). The selected channels had a smaller neuromechanical coupling factor and a smaller bias than the channels with the lower SNR value ($p=0.27$ and $p<.001$, respectively). For this reason and taking into account the improved correlation with $\mathrm {PTP}_\mathrm {mus}$, we proceed to employ $\mathrm {ETP}_\mathrm {sel}$ as the sEMG-based measure for the inspiratory effort.

**Table 3:** Neuromechanical conversion factors and biases of sEMG-derived effort metrics against ${\mathrm{PTP}}_{\mathrm{mus}}$, $n=41$

	${\mathrm{ETP}}_{\mathrm{di}}$	${\mathrm{ETP}}_{\mathrm{interc}}$	${\mathrm{ETP}}_{\mathrm{sel}}$
$\textit{K}_{\mathrm{EMG}}$ ($\hbox {cm}\hbox {H}_{2}\hbox {O}/\upmu \hbox {V}$)	4.48 ± 3.89	4.71 ± 4.07	4.32 ± 3.73
$\textit{P}_{\mathrm{bias}}$ (${\hbox {cm}\hbox {H}_{2}\hbox {O}}$)	1.38 ± 1.63	1.16 ± 2.16	0.69 ± 1.43

Both parameters ($\textit{K}_{\mathrm{EMG}}$ and $\textit{P}_{\mathrm{bias}}$) were determined by fitting the different ETP metrics to ${\mathrm{PTP}}_{\mathrm{mus}}$ via the linear regression model in Eq. (1). The parameter $\textit{P}_{\mathrm{bias}}$ represents systemic offsets of the EMG signal against ${\mathrm{P}}_{\mathrm{mus}}$

We tested the linearity of the EMG-$P_{\mathrm {mus}}$ relation by also fitting two nonlinear models to the $\mathrm {ETP}_\mathrm {sel}$-$\mathrm {PTP}_\mathrm {mus}$ data (visualized in the scatter plot in Fig. 4) and comparing it to the linear model in Eq. (1). The two nonlinear models had an additional quadratic term and an additional square root term, respectively. For all three models, we calculated the adjusted coefficient of determination $r^2_\mathrm {adj}$. We found a significant ($p<.001$) but small difference between the linear model ($r^2_\mathrm {adj}={0.79\pm 0.13}$) and the two nonlinear models ($r^2_\mathrm {adj}={0.80\pm 0.12}$ and $r^2_\mathrm {adj}={0.81\pm 0.10}$), concluding that the assumption of linearity is viable in our patient cohort and over the studied range of activities.

**Fig. 5:**
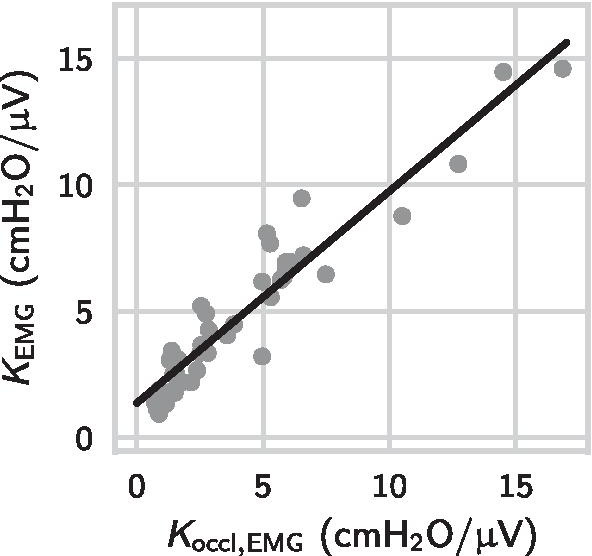
Correlation between neuromechanical conversion factor determined during multiple subsequent occlusions $\textit{K}_{\mathrm{occl,EMG}}$ and the reference value $\textit{K}_{\mathrm{EMG}}$ determined by directly fitting the selected EMG channel (${\mathrm{ETP}}_{\mathrm{sel}}$) to ${\mathrm{PTP}}_{\mathrm{mus}}$. Each point represents one patient

As a next step, we investigate the possibility to estimate $K_\mathrm {EMG}$ during occlusions. The two scalars $K_\mathrm {EMG}$ and $K_\mathrm {occl,EMG}$ were highly correlated ($r=0.95$, $p<.001$, $\text {slope}=0.84$), cf. Fig. 5. The ratio $K_\mathrm {EMG}/ K_\mathrm {occl,EMG}$ was ${0.81\pm 0.23}$, which indicates a systematic overestimation of the neuromechanical conversion factor determined during occlusions, which was also previously recognized by [12, 22] and prescribed to the changed configuration of the diaphragm during occlusions. Therefore, in Eq. (3), we use the correction factor $\alpha = 0.8$ to account for the deviation, (which coincides with the correction factor given by [12, 22], who proposed 1/1.25).

#### Inspiratory effort estimation

**Fig. 6:**
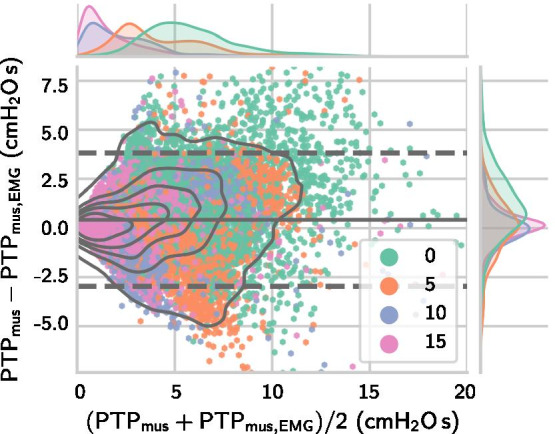
Bland-Altman plot for sEMG-derived ${\mathrm{PTP}}_{\mathrm{mus,EMG}}$ via Eq. (3) against ${\mathrm{PTP}}_{\mathrm{mus}}$. The plot depicts $\textit{m}= 18{\,}341$ efforts from $\textit{n}= {41}$ patients, each point represents one breath. The limits of agreement were calculated using the Bland-Altman method for repeated measurements, cf. [31]. The mean and ${95}\%$ interval are visualized via the solid grey line and dashed grey lines, respectively

**Fig. 7:**
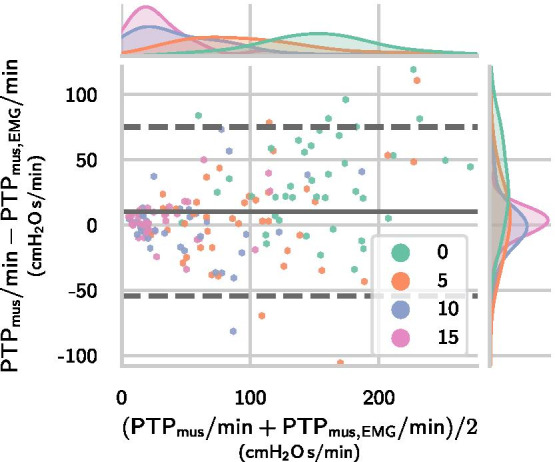
Bland-Altman plot for sEMG-derived ${\mathrm{PTP}}_{\mathrm{mus,EMG}}/\hbox {min}$ via Eq. (3) against ${\mathrm{PTP}}_{\mathrm{mus}}/\hbox {min}$. The plot depicts PTP$/\hbox {min}$ values for $\textit{n}= {41}$ patients and in each patient one point per pressure support level is plotted, i.e., four points for each patient. The limits of agreement were calculated using a variant of the Bland-Altman method for repeated measurements, cf. [31]. The mean and ${95}\%$ interval are visualized via the solid grey line and dashed grey lines, respectively

The breath-wise deviation between the sEMG-derived measure $\mathrm {PTP}_\mathrm {mus,EMG}$ and the $P_{\mathrm {es}}$-derived reference $\mathrm {PTP}_\mathrm {mus}$ was calculated across all efforts from all datasets containing long occlusions ($m=18\,341$ efforts and $n=41$ patients). As the data included multiple measurements from each patient and substantially different numbers of breaths, the mean and standard deviation of differences were calculated using a variant of the classical Bland-Altman method accounting for repeated measures [31]. The breath-wise deviation between $\mathrm {PTP}_\mathrm {mus,EMG}$ and $\mathrm {PTP}_\mathrm {mus}$ was ${0.43\pm 1.73}\,{\hbox {cm}\hbox {H}_{2}\hbox {O}\,\hbox {s}}$ and the MAD was ${1.23}\,{\hbox {cm}\hbox {H}_{2}\hbox {O}\,\hbox {s}}$. As a last step, we evaluated the deviation between $\mathrm {PTP}_\mathrm {mus,EMG}/\hbox {min}$ and $\mathrm {PTP}_\mathrm {mus}/\hbox {min}$ values calculated by aggregating all efforts within each pressure support level and found an error of ${10.3\pm 33.0}\,{\hbox {cm}\hbox {H}_{2}\hbox {O}\,\hbox {s}/\hbox {min}}$ and an MAD of ${23.9}\,{\hbox {cm}\hbox {H}_{2}\hbox {O}\,\hbox {s}/\hbox {min}}$. The Bland-Altman plots in Figs. 6 and 7 show that the occlusion-based estimator provides approximations to the inspiratory effort within clinically acceptable bounds and with a small bias.

## Discussion

Our results indicate that surface EMG of the respiratory muscles can be measured reliably and, using our proposed methodology, serves to non-invasively monitor the breath-by-breath inspiratory effort in mechanically ventilated patients. Our main findings can be summarized as follows. Firstly, the sEMG-time product (ETP) of the selected channel is well correlated with $\mathrm {PTP}_\mathrm {mus}$ (and $\mathrm {PTP}_\mathrm {di}$) calculated from esophageal/gastric pressure and has only a small bias against $\mathrm {PTP}_\mathrm {mus}$. Secondly, using a linear neuromechanical conversion factor determined during multiple occlusions, the sEMG-derived breath-by-breath pressure-time product $\mathrm {PTP}_\mathrm {mus,EMG}$ can be used to estimate $\mathrm {PTP}_\mathrm {mus}$ with an acceptably small error across a large cohort of patients scheduled for bronchoscopy. Thirdly, the benefit of multiple sEMG channels can be leveraged via a channel selection method. In clinical practice, the measurement of patient efforts via sEMG is highly attractive for assessment of inspiratory effort and $\mathrm {PTP}_\mathrm {mus}$ as it is non-invasive and does not require the placement of esophageal probes as EAdi and $P_{\mathrm {es}}$ do.

The main goal of this work was to investigate the quantification of inspiratory effort through sEMG-derived $P_{\mathrm {mus,EMG}}$: to this end, we have reported that the deviation of $\mathrm {PTP}_\mathrm {mus,EMG}$ to $\mathrm {PTP}_\mathrm {mus}$ calculated on a breath-by-breath basis was ${0.43\pm 1.73}\,{\hbox {cm}\hbox {H}_{2}\hbox {O}\,\hbox {s}}$ and that the mean absolute deviation was ${1.23}\,{\hbox {cm}\hbox {H}_{2}\hbox {O}\,\hbox {s}}$ on a large cohort of patients scheduled for bronchoscopy. We believe that this accuracy would be acceptable in clinical practice, and that the proposed method thus enables continuous, non-invasive assessment of patients’ inspiratory efforts. The deviation between $\mathrm {PTP}_\mathrm {mus,EMG}/\hbox {min}$ and $\mathrm {PTP}_\mathrm {mus}/\hbox {min}$ values calculated within each support level was ${10.3\pm 33.0}\,{\hbox {cm}\hbox {H}_{2}\hbox {O}\,\hbox {s}/\hbox {min}}$, which is quite low when compared to a clinical value range of ${0{-}250}\,{\hbox {cm}\hbox {H}_{2}\hbox {O}\,\hbox {s}/\hbox {min}}$ (and a target range of ${100{-}150}\,{\hbox {cm}\hbox {H}_{2}\hbox {O}\,\hbox {s}/\hbox {min}}$). The herein reported accuracy may help to promote the application of sEMG in clinical practice. We have also reported a small bias (see $P_{\mathrm {bias}}$) of breath-wise ETP values fitted to $\mathrm {PTP}_\mathrm {mus}$ by means of linear regression and we found a small bias in the deviation between $\mathrm {PTP}_\mathrm {mus,EMG}$ and $\mathrm {PTP}_\mathrm {mus}$. We found the correction for offsets in the EMG envelopes to play a crucial role in achieving a small bias between ETP and $\mathrm {PTP}_\mathrm {mus}$. For this offset correction, we used an adaptive, time-varying baseline.

Our findings corroborate existing evidence for the validity of respiratory EMG (either measured from the esophagus or transcutaneously) for quantifying the inspiratory effort of patients under assisted ventilation. Beck et al. [29] observed a strong correlation ($r={0.84\pm 0.12}$) between EAdi and $P_{\mathrm {di}}$ in patients under assisted ventilation. A similarly high correlation was later reported by Bellani et al. [12] between EAdi and the total respiratory muscle pressure $P_{\mathrm {mus}}$. First encouraging results regarding the correlation between diaphragmatic *surface* EMG and $P_{\mathrm {mus}}$ were then reported by Bellani et al. [22]. Their analysis however relied on the reduction of measurement noise trough aggregation of similar breaths. This study is the first to show a high correlation between the values of the breath-wise sEMG-time product and the $P_{\mathrm {mus}}$-time product studied over a wide range of patient activities without relying on any aggregation of multiple breaths. Thus, building upon the analysis by Bellani et al. [22], we provide first empirical evidence for the feasibility of breath-by-breath effort quantification via sEMG. We also found that the sEMG-$P_{\mathrm {mus}}$ relation can be well approximated using a linear model and that nonlinear models do not provide a substantially better representation of the data.

Furthermore, we have provided further evidence for the validity of the occlusion-based method for determining the neuromechanical conversion factor $K_\mathrm {EMG}$. Consistent with [12], we found a systematic overestimation of the factor determined during occlusions and a similar magnitude for this effect: we also found a correction factor of 0.8 to be a good choice in most patients. The systematic overestimation can be explained through the more favourable configuration of the muscles during isometric contraction and their force–length relationship: respiratory muscles attain an increased neuromechanical efficiency at end-expiration compared to higher lung volumes, at which the muscles are shortened [32, 33].

As with any physiological measurement, obtaining a signal with a high signal-to-noise ratio (SNR) is crucial when working with respiratory sEMG measurements. Several factors influence the SNR of such a measurement, including (1) the activation patterns of the diaphragm and intercostal muscles, (2) the geometry and conductivity of the biological tissues separating muscle fibers and recording electrodes and (3) the level of measurement noise at different electrodes due to physiological and non-physiological interference. A good SNR in either one of the channels therefore corresponds to a favourable measurement condition, i.e., substantial muscle activation with a good transmission of the EMG signal to the electrode at a low level of noise. In this work, we have demonstrated that a simple, approximate SNR-based channel selection method can substantially increase the SNR of the resulting measurement and thereby increase the correlation of ETP to both $\mathrm {PTP}_\mathrm {mus}$ and $\mathrm {PTP}_\mathrm {di}$. This is in contrast to earlier studies, where different channel combination techniques have been tested that did not lead to an improved correlation to $P_{\mathrm {mus}}$ [22] . Our results support the merit of using multiple sEMG channels to capture respiratory activity from different muscle groups and carefully selecting the channel with the more favourable measurement condition.

It is currently an open question whether the ratio of the signal amplitudes observed in the two sEMG channels has additional diagnostic value. It is well known that muscle activation might shift from the diaphragm to accessory muscles under high-stress conditions [22, 34]. However, it is not yet clear whether this effect can be reliably observed via sEMG. We have reported a high correlation between the diaphragmatic and intercostal sEMG channels ($r={0.74\pm 0.27}$), which might indicate that muscle recruitment did not change throughout the protocol. This hypothesis is corroborated by the very high correlation of $P_{\mathrm {di}}$ and $P_{\mathrm {mus}}$. In our study, the signal amplitudes in the intercostal channel were large and estimated SNR of this channel was higher than that of the diaphragmatic channel. (Thus, the intercostal channel was selected more often as the more informative channel.) We therefore hypothesize that this channel contains important information about the total inspiratory effort of the patient. The neuromechanical conversion factors $K_\mathrm {EMG}$ for sEMG varied widely between patients, which was also previously reported for EAdi [22]. A particular level of sEMG amplitude can thus correspond to a wide range of generated muscle pressures, and the absolute value of the measured sEMG should therefore be interpreted cautiously.

When comparing $P_{\mathrm {es}}$-derived measures of inspiratory effort with surrogate measures (EAdi or EMG), it should always be taken into account that $P_{\mathrm {es}}$ itself is subject to measurement errors. These errors may result from, e.g., peristalsis, cardiac artifacts or incorrect catheter positioning. In our study, we have attempted to mitigate the influence of cardiac interference in the $P_{\mathrm {es}}$ signal by using our previously described template subtraction method [25]. Moreover, we have corrected any scaling errors in the $P_{\mathrm {es}}$-derived $P_{\mathrm {mus}}$ reference signal using a factor $K_\mathrm {occl,es}$ determined by fitting $P_{\mathrm {es}}$ directly to the $P_{\mathrm {aw}}$ signal during multiple subsequent occlusions. We believe that this additional scaling correction helps to reduce the influence of esophageal balloon positioning errors. Nevertheless, one should use this approach with caution and only if multiple subsequent occlusions are available and the slope between $P_{\mathrm {es}}$ and $P_{\mathrm {aw}}$ is already close to one.

Several limitations of our study must be acknowledged. Most of the included patients had no severe acute or chronic respiratory failure despite preexisting pulmonary diseases, and patients were not ventilated over a prolonged period of time. This might limit the applicability of our results to the intensive care setting, since breathing patterns in those patients differ from those in our population. Moreover, as this study lacks patients with BMI $>{35}\,{\hbox {kg}/\hbox {m}^2}$, the body weight (cf. Table 1) was not fully representative for intensive care units, where obesity is increasingly becoming a common comorbidity. Also, thoraco-abdominal surgery might alter the surface recording of respiratory muscles and was not addressed in this work. Therefore, further studies will be needed to demonstrate the reliability of sEMG recordings for quantifying inspiratory effort in the intensive care setting. Finally, we have studied a relatively wide range of respiratory muscle activity and have also included fully spontaneous breathing under CPAP which has to be taken into account when comparing the reported correlations. The studied range of activities was considerably larger than that of Bellani et al. [12], who included ${\pm \,4}\,{\hbox {cm}\hbox {H}_{2}\hbox {O}}$ pressure support from their baseline support level, but similar to the range studied by Beck et al. [29], who also included CPAP in most of their patients.

## Conclusions

The current clinical gold standard for measuring inspiratory effort, $P_{\mathrm {es}}$, is invasive and prone to recording artifacts and positioning errors. For these reasons, despite its clinical importance, monitoring of respiratory effort is still not a standard procedure in many intensive care units. Our results support the use of surface electromyography as a non-invasive alternative for monitoring the inspiratory effort of patients and may help to promote its application in clinical practice.

## Supplementary Information

**Additional file 1.** Signal processing details: filtering of esophageal and gastric pressures, determination of chest wall elastance, automatic detection of efforts and sEMG offset correction.

**Additional file 2.** Mean and standard deviation of PTP/min, ETP/min, minute ventilation, and dynamic intrinsic PEEP within each pressure support level across all patients.

## Data Availability

The datasets used and/or analyzed during the current study are available upon reasonable request, provided approval is granted by the ethics committee of the Witten/Herdecke University (Witten, Germany).

## Abbreviations

ACOS: Asthma-COPD overlap syndrome
BMI: Body-mass index
COPD: Chronic obstructive pulmonary disease
CPAP: Continuous positive airway pressure
EAdi: Electrical activity of the diaphragm
EMG: Electromyography
ETP: EMG-time product
FEV1: Forced expiratory volume in 1 s
ILD: Interstitial lung disease
MAD: Mean absolute deviation
OSAS: Obstructive sleep apnea syndrome
PEEP: Positive end-expiratory pressure
PSV: Pressure support ventilation
PTP: Pressure-time product
RV: Residual volume
sEMG: Surface electromyography
SNR: Signal-to-noise ratio
SpO2: Peripheral oxygen saturation
TLC: Total lung capacity
VC: Vital capacity
WOB: Work of breathing
