# Supplementary material for: Surface EMG-based quantification of inspiratory effort: a quantitative comparison with Pes
Source: Crit Care. 2021 Dec 20;25:441. doi: 10.1186/s13054-021-03833-w (PMC8686581; doi:10.1186/s13054-021-03833-w)
Supplement: Supplementary file 1 — Additional file 1. Signal processing details: filtering of esophageal and gastric pressures, determination of chest wall elastance, automatic detection of efforts and sEMG offset correction. [file 13054_2021_3833_MOESM1_ESM.pdf]

Signal Processing Details

Processing of P_{es} and P_{ga}

Volume Signal V

A continuous lung volume signal V was generated by calculating the running integral over the airflow measurement. Due to small flow sensor inaccuracies or leakages, this integration usually results in long-term drifts in the estimated volume signal. To correct for these drifts, we applied a baseline removal algorithm to the end-expiratory volumes (assuming that on average over multiple breaths the end-expiratory volume is zero). Briefly, end-expiratory volumes were filtered via a moving median, a continuous baseline was calculated by interpolating between these filtered points and then the baseline was subtracted from the volume signal.

Cardiac Artifact Removal

Prior to further analysis, cardiogenic pressure artifacts were removed from both the P_{ga} and P_{es} signals. For this step, a template subtraction method was employed, cf. [1] for details. Any remaining artifacts are then removed from P_{es} and P_{ga} using a low-pass filter (5th order Butterworth filter with 7 Hz cutoff).

Determination of E_{cw}

Next, the elastic chest wall recoil was analyzed during a phase with the highest level of pressure support ($15\,\mathrm{cmH_2O}$) and low overall patient activity. Since there were no phases without residual spontaneous breathing activity, special care was taken to select pairs of data points (each pair consisting of one point at the end of expiration and one point at the end of the subsequent inspiration) that were not affected by patient efforts. The validity of the selected data points was verified by careful manual inspection of the P_{es} and P_{ga} waveforms. The chest wall elastance E_{cw} was then calculated in the Campbell diagram as the slope between the annotated points and then averaged over multiple annotated (semi-passive) breaths for each patient. An exemplary segment of data is provided in figure 1.

Muscular and Transdiaphragmatic Pressure

The time course of the transdiaphragmatic pressure P_{di} was then calculated as the difference between P_{ga} and P_{es}:

$$P_{di} = P_{ga} - P_{es}. \tag{1}$$

The pressure P_{mus} generated by the respiratory muscles at each instant was calculated as the difference between esophageal pressure P_{es} and the elastic recoil of the chest wall, $P_{cw} = E_{cw} \cdot V$:

$$P_{mus} = E_{cw} \cdot V - P_{es}. \tag{2}$$

Finally, the absolute value of P_{es} strongly depends on the filling and positioning of the esophageal balloon [2]. For this reason, throughout the further analyses we only evaluate pressure swings of P_{di} and P_{mus} relative to a *baseline* which was determined during passive expirations.

Automatic Detection of Efforts

Signals were segmented into inspirations and expirations using a simple, threshold-based detector that was directly applied to the P_{mus} signal. The detector was based on the trigger algorithm proposed by Sinderby et al. [3]. To detect the onset of inspiration, a threshold of $0.5\,\mathrm{cmH_2O}$ was used and the onset of expiration was detected at the point where P_{mus} had decreased to $70\,\%$ of its inspiratory maximum. We also applied the defragmentation approach proposed by Sinderby et al. [3]: to this end, all detected breaths shorter than $0.3\,\mathrm{s}$ were discarded as invalid and subsequent breaths that were within $0.35\,\mathrm{s}$ of each other were merged together.

Processing of sEMG Signals

sEMG Envelope Correction

The offsets of sEMG signals were corrected by calculating a time-varying baseline. Empirically, we found the first tercile of the envelope signal within a moving $5\,\mathrm{s}$ window to be a robust baseline estimator (i.e., one third of the envelope values within this window are smaller then the baseline value). This baseline was calculated individually for both available channels and then subtracted from the envelopes. Exemplary data is provided in figure 2.

Automatic Channel Selection

The SNR of the two sEMG channels was approximated by comparing the maximum amplitudes reached during tidal breathing with the amplitude of the measurement noise. To this end, the distributions of the (not baseline-corrected) sEMG envelopes were analyzed by forming the ratio

$$\mathrm{SNR_{approx}} = \frac{Q_3}{Q_1}, \tag{3}$$

with Q_3 denoting the third quartile (quantifying the EMG amplitudes reached during active efforts) and Q_1 denoting the first quartile (quantifying the level of noise). The running value of $\mathrm{SNR_{approx}}$ was calculated over a moving $10\,\mathrm{s}$ window. Finally, for each patient the channel with the higher median $\mathrm{SNR_{approx}}$ over the whole recording was selected for quantifying inspiratory effort. The selected envelope signal is denoted as $\mathrm{EMG_{sel}}$.

Exemplary Data: E_{cw} Determination

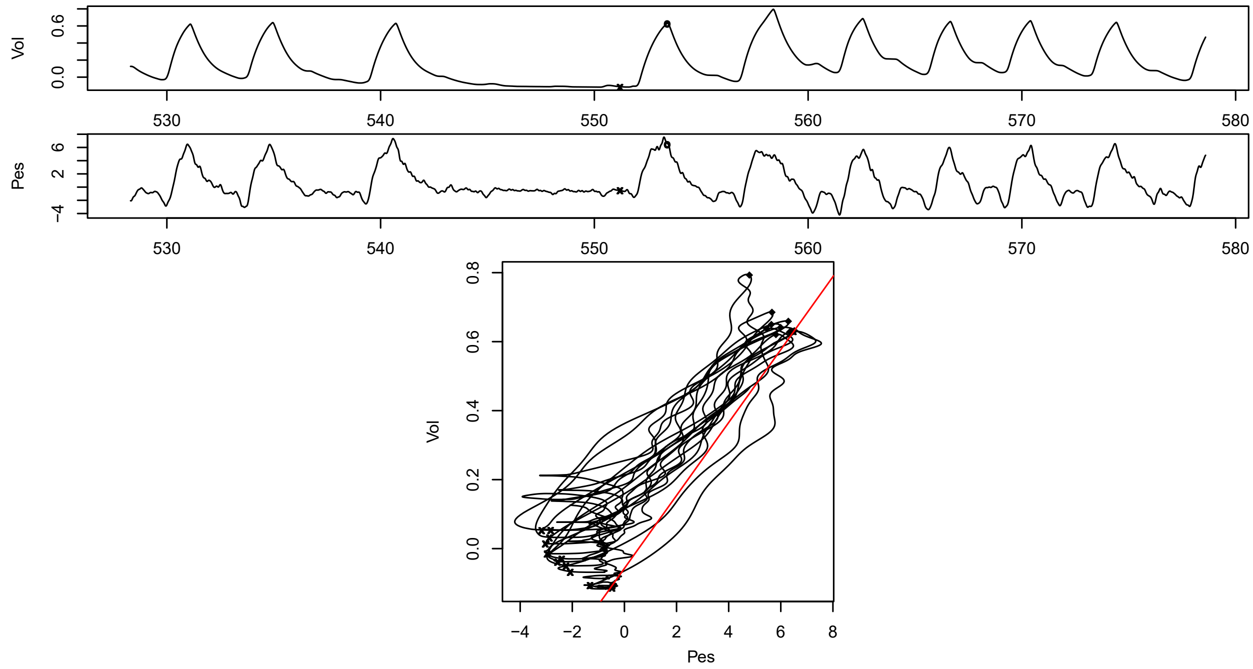

Figure 1 Segment of data with 15 cmH_2O of pressure support. The patient is active (this can be seen from the negative deflections in P_{es}). A breath is selected where the disturbance by the patient is minimal and end-expiratory & end-inspiratory points are used for determing chest wall elastance E_{cw} (see markers in the two upper graphs). E_{cw} is calculated from the slope between the marked points in the Campbell diagram (red line in the bottom graph).

Exemplary Data: sEMG Baseline

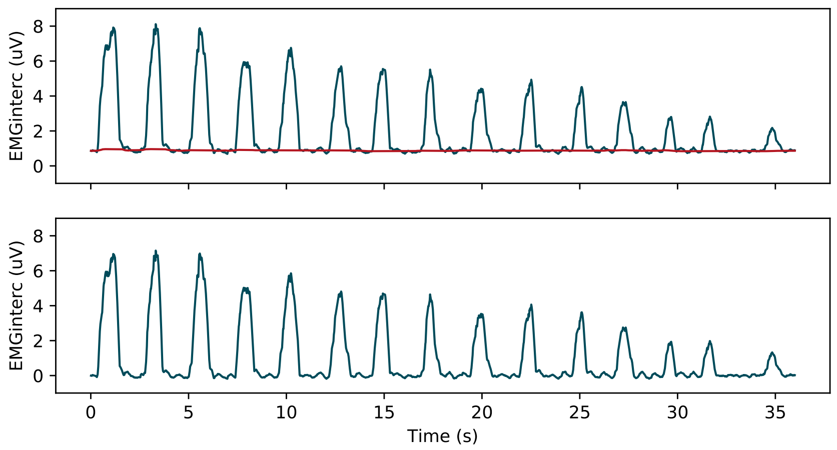

Figure 2 Segment of data showcasing the baseline correction of sEMG envelopes. The upper graph shows the envelope of the intercostal EMG (green line) and the adaptive baseline (red line). The bottom graph shows the difference between the two, i.e. the baseline corrected envelope.

References

1. Graßhoff J, Petersen E, Eger M, Bellani G, Rostalski P. A Template Subtraction Method for the Removal of Cardiogenic Oscillations on Esophageal Pressure Signals. In: Proceedings of the 39th Annual International Conference of the IEEE Engineering in Medicine and Biology Society (EMBC). New Jersey, USA: IEEE Engineering in Medicine and Biology Society; 2017. p. 2235—-2238.
2. Mauri T, Yoshida T, Bellani G, Goligher EC, Carteaux G, Rittayamai N, et al. Esophageal and transpulmonary pressure in the clinical setting: meaning, usefulness and perspectives. Intensive Care Medicine. 2016 September;42(9):1360—–1373.
3. Sinderby C, Liu S, Colombo D, Camarotta G, Slutsky AS, Navalesi P, et al. An automated and standardized neural index to quantify patient-ventilator interaction. Critical Care. 2013;17:R239.
