# Supplementary material for: Surface EMG-based quantification of inspiratory effort: a quantitative comparison with Pes
Source: Crit Care. 2021 Dec 20;25:441. doi: 10.1186/s13054-021-03833-w (PMC8686581; doi:10.1186/s13054-021-03833-w)
Supplement: Supplementary file 2 — Additional file 2. Mean and standard deviation of PTP/min, ETP/min, minute ventilation, and dynamic intrinsic PEEP within each pressure support level across all patients. [file 13054_2021_3833_MOESM2_ESM.pdf]

	PTP_{mus}/min (cmH$_2$0 s/min)	PTP_{aw}/min (cmH$_2$0 s/min)	ETP_{di}/min (μV s/min)	ETP_{interc}/min (μV s/min)	ETP_{sel}/min (μV s/min)	MV (L/min)	iPEEP (cmH$_2$0)	$PTP_{mus,EMG}$/min (cmH$_2$0 s/min)
**CPAP**	170.2 ± 54.8	2.5 ± 1.6	42.9 ± 32.7	52.0 ± 54.1	52.1 ± 41.0	7.7 ± 1.9	2.1 ± 1.4	137.4 ± 47.6
**PS 5**	101.9 ± 60.2	65.2 ± 9.4	29.4 ± 24.5	44.7 ± 85.4	37.3 ± 35.0	7.6 ± 1.8	2.2 ± 1.7	98.1 ± 53.2
**PS 10**	48.2 ± 44.5	118.2 ± 25.9	13.7 ± 15.1	23.4 ± 67.9	17.1 ± 21.6	7.0 ± 1.8	1.8 ± 1.7	48.4 ± 41.8
**PS 15**	33.8 ± 30.7	143.3 ± 34.2	8.4 ± 7.5	26.3 ± 88.5	10.0 ± 8.9	6.5 ± 1.7	1.5 ± 1.5	30.3 ± 26.7
